# Longitudinal patterns of postoperative brain oxygen saturation in children with congenital heart disease and postoperative delirium risk: a repeated measures analysis

**DOI:** 10.3389/fped.2026.1837715

**Published:** 2026-06-22

**Authors:** Yingzi Xie, Li Yuan, Xinlan Wu

**Affiliations:** Department of Cardiothoracic Surgery, Shanghai Children’s Medical Center, School of Medicine, Shanghai Jiao Tong University, Shanghai, China

**Keywords:** cardiopulmonary bypass, cerebral oxygen saturation, congenital heart disease, pediatric cardiac surgery, postoperative delirium

## Abstract

**Objective:**

To investigate longitudinal patterns of regional cerebral oxygen saturation (CrSO_2_) after cardiopulmonary bypass (CPB) surgery in children with congenital heart disease (CHD) and their association with postoperative delirium (POD).

**Methods:**

A prospective longitudinal study was conducted involving 65 pediatric patients undergoing corrective cardiac surgery with CPB. CrSO_2_ level were monitored at 6 h (T1), 12 h (T2), 24 h (T3), and 48 h (T4) postoperatively. POD was assessed using the Cornell Assessment of Pediatric Delirium (CAPD). Multivariate logistic regression and linear mixed-effects models (LMMs) were applied to analyze CrSO2 trajectories and their association with POD.

**Results:**

The incidence of POD was 47.7% (31/65). Univariate analysis showed significantly lower CrSO_2_ levels at T1-T4 and minimum CrSO_2_ values in the POD group compared to the NPOD group (all *P* < 0.05). Multivariate logistic regression indicated that lower CrSO_2_ levels in the early postoperative period independently predicted POD (OR = 0.941, 95% CI: 0.894–0.991, *P* = 0.021). The model exhibited good fit (*P* = 0.037) and passed the Hosmer–Lemeshow test (*P* = 0.132). LMM analysis revealed significantly lower CrSO_2_ levels in the POD group at 6 h, 12 h, and 48 h postoperatively (Bonferroni-corrected *P*-values: 0.009, 0.030, and 0.018, respectively), although the overall CrSO_2_ trend over time was similar between groups.

**Conclusion:**

Early postoperative CrSO_2_ dynamics in pediatric CHD patients undergoing CPB are independently associated with POD. Children in the POD group exhibited consistently lower CrSO_2_ levels, especially at 6, 12, and 48 h postoperatively. Continuous monitoring of CrSO_2_ could facilitate early detection and timely intervention for POD.

## 1 Introduction

Congenital heart disease (CHD) is the most common congenital malformation (1), and surgical correction remains the cornerstone for improving patient prognosis (2). However, cardiac surgery involving cardiopulmonary bypass (CPB) exposes the central nervous system to multiple risks, such as ischemia-reperfusion injury and inflammatory responses, potentially triggering various neurological complications (3). Among these, postoperative delirium (POD), an acute, fluctuating neuropsychiatric syndrome (4), has emerged as a significant clinical challenge affecting both short- and long-term outcomes in pediatric patients.

Epidemiological data indicate a high incidence of pediatric cardiac POD, ranging from 49% to 59.1% (5). POD not only increases the risk of brain injury but is also closely associated with extended durations of mechanical ventilation, ICU stay, and total hospitalization, thereby exacerbating the healthcare burden. In the long term, POD may cause persistent impairments in children's cognitive function and motor coordination (6). More critically, pediatric POD often presents with transient and atypical symptoms (7), making it prone to being overlooked in the busy ICU environment, thereby missing critical opportunities for early intervention.

Against this backdrop, early postoperative noninvasive, continuous monitoring of brain function has become a research focus. Near-infrared spectroscopy (NIRS)-based monitoring of regional cerebral tissue oxygen saturation (CrSO_2_) provides a real-time indication of the balance between cerebral oxygen supply and demand. Previous studies have shown that intraoperative and postoperative reductions in CrSO_2_ are significantly associated with adverse neurological outcomes (8, 9). Furthermore, CrSO_2_ min and postoperative CrSO_2_ levels have been associated with POD (10). However, most existing studies are based on cross-sectional or single-time-point data and fail to capture the continuous dynamic evolution of early postoperative cerebral oxygenation and its independent association with POD. In addition, there is a lack of systematic characterization of the longitudinal trajectory of CrSO_2_ in the early postoperative period following CPB in children with CHD. Given that CrSO_2_ is a physiological indicator that fluctuates over time, its longitudinal patterns may contain richer prognostic information than single measurements. This critical knowledge gap has limited a comprehensive understanding of the mechanisms underlying POD from a dynamic perspective.

To address this gap, this study was designed as a prospective longitudinal observational study. Clinical and monitoring data were systematically collected within 48 h postoperatively from pediatric patients who underwent CPB-assisted CHD repair at our hospital between November 2024 and November 2025. The core methodology and innovation of this study lie in the first application of a linear mixed-effects model (LMM) in this population to characterize longitudinal patterns of early postoperative CrSO_2_ and to analyze its relationship with POD. We evaluated whether CrSO_2_ was independently associated with POD using three approaches: between-group comparisons, analysis of longitudinal changes, and multivariate logistic regression. This multidimensional, dynamic analytical framework overcomes the limitations of traditional cross-sectional studies and provides a more precise theoretical basis for time window-specific individualized brain protection and early detection of POD.

## 2 Article types

This manuscript is an original research article. It reports the results of a prospective longitudinal clinical study designed to investigate the patterns of postoperative regional CrSO_2_ in children with CHD undergoing CPB, as well as its association with the risk of POD.

## 3 Materials and methods

### 3.1 Study population

This prospective longitudinal analysis included pediatric patients who underwent corrective surgery for CHD with CPB at Shanghai Children's Medical Center between November 2024 and November 2025. Inclusion criteria were as follows: (1) Diagnosis of CHD confirmed by color doppler echocardiography; (2) Age 0–18 years; (3) No severe neurological or metabolic complications at diagnosis; (4) Informed consent obtained from guardians. Exclusion criteria were as follows: (1) Chromosomal abnormalities or impaired brain function (e.g., head trauma, intracranial lesions); (2) History of preoperative resuscitation; (3) Severe perioperative adverse events or death.

This study protocol was reviewed and approved by the Institutional Review Board of the Shanghai Children's Medical Center, affiliated with the School of Medicine at Shanghai Jiao Tong University, on September 9, 2024 (Approval No. SCMCIRB-K2024156-1). Following approval by the Ethics Committee, patients who met the inclusion criteria were enrolled prior to surgery. Written informed consent was obtained from the parents or legal guardians of all participants to data collection. The postoperative monitoring protocol was predefined in the study protocol and was prospectively applied to all enrolled subjects.

### 3.2 Research methods

#### 3.2.1 Clinical data collection

Clinical data were systematically retrieved from the hospital's electronic medical record system and surgical anesthesia records, including: (1) General demographic characteristics: gender, age (months), height (cm), weight (kg), and primary diagnosis after surgery. (2) Perioperative variables: CPB duration (min), aortic cross-clamp time (min), and total surgery duration (min).

#### 3.2.2 Observation indicators and monitoring methods

All patients were transferred to the Cardiac and Thoracic Intensive Care Unit (CICU) for continuous monitoring after surgery.

Regional CrSO_2_ monitoring: using a NIRS monitor (Mindray), the probe (CNN) was secured to the child’s forehead and CrSO_2_ values were recorded at T1-T4 (6, 12, 24, and 48 h postoperatively).

Arterial blood gas parameters: Arterial blood samples were collected for blood gas analysis concurrently with CrSO_2_ measurements (6, 12, 24, and 48 h postoperatively). Arterial oxygen saturation (SaO_2_) and lactate levels were recorded.

Clinical prognosis indicator: CICU length of stay (hours) during the index hospitalization was recorded.

Other parameters: Mean arterial pressure (MAP) from T1 to T4, maximum vasoactive-inotropic score (VISmax) within the first 24 h postoperatively, and VISmax within the second 24 h postoperatively were documented.

#### 3.2.3 Assessment criteria for relevant scales

All scale assessments were performed by CICU physicians or nurses who had received standardized training. Assessments were conducted approximately 30 min after extubation and in the absence of sedation to ensure objectivity and consistency.

Sedation and agitation assessment: The Richmond Agitation-Sedation Scale (RASS) (11) was used to evaluate the level of consciousness. Scores range from −5 (deep sedation) to +4 (combative behavior), with 0 indicating an alert and calm state. Negative values indicate sedation, whereas positive values indicate agitation. Higher absolute scores reflect more severe impairment of consciousness.

POD assessment: The Cornell Assessment of Pediatric Delirium (CAPD) (12) was used for screening. This 8-item scale evaluates eye contact, purposeful actions, awareness of surroundings, agitation, response to comfort, activity level, responsiveness, and communication needs of the child and caregiver. Each item is scored from 0 to 4, resulting in a total score ranging from 0 to 32. with scores ≥ 10 indicating POD. CAPD assessments were performed only when the RASS score was ≥ −3 to rule out interference from deep sedation. If a child's RASS score was < −3, it was recorded as “deeply sedated, not assessed,” and CAPD screening commenced at the first assessment after the RASS score returned to ≥ −3. Prior to planned extubation, all pediatric patients followed the department's standard weaning protocol: sedative medication infusions were gradually reduced and discontinued, and endotracheal tubes were removed only after patients regained consciousness and reached a clinically assessable state (RASS ≥ −3). Therefore, CAPD assessments conducted after extubation ensured that all patients met the required level of consciousness for reliable evaluation.

Vasopressor Index (VIS) (13) = Dopamine dose [μg/(kg·min)] + Dobutamine dose [μg/(kg·min)] + 100 × Epinephrine dose [μg/(kg·min)] + 100 × Norepinephrine dose [μg/(kg·min)] + 10,000 × Vasopressin dose [U/(kg·min)] + 10 × Milrinone dose [μg/(kg·min)]. Higher VIS values indicate greater dependence on vasoactive support.

#### 3.2.4 Sample size calculations and *post-hoc* efficacy analysis

The initial sample size was determined primarily based on clinical feasibility and experience, and no formal *a priori* sample size calculation was performed. To assess the statistical power of the primary between-group comparison in this study, a *post-hoc* analysis comparing CrSO_2_ levels at 6 h postoperatively between the POD and NPOD groups was performed. Using G*Power 3.1, the calculated statistical power (1-*β*) was approximately 0.70.

### 3.3 Statistical analysis

Statistical analysis was performed using SPSS version 26.0, with a significance level of *α* = 0.05.

1. Descriptive analysis: Categorical variables were described using frequencies (n) and proportions (%). Continuous variables were tested for normality; variables with normal distribution were expressed as mean ± standard deviation (SD), while those with skewed distribution were expressed as median and interquartile range (IQR).
2. Intergroup comparisons: Baseline characteristics between the POD and NPOD groups were compared using *χ*^2^ or Fisher's exact tests for categorical variables; two-sample t-tests for normally distributed continuous variables; and Mann–Whitney U tests for skewed continuous variables.
3. Multivariate logistic regression analysis: With POD as the dependent variable, a main model and sensitivity models were constructed. The main model included CrSO2 at T1, VISmax within the first 24 h postoperatively, lactate at T1, and age (months) as independent variables. To assess the robustness of these results, sensitivity analyses were performed, additionally controlling for CrSO2 at T1, postoperative CrSO2 min, and postoperative mechanical ventilation duration. Results are reported as odds ratios (OR) with 95% confidence intervals (CI). Model fit was evaluated using the Hosmer–Lemeshow test.
4. Longitudinal repeated-measures analysis: LMM were used to analyze dynamic CrSO2 changes. The baseline model included time, POD grouping, and their interaction (time × POD) as fixed effects, with individual patients as random intercepts and a first-order autoregressive (AR1) covariance structure for repeated measures. An adjusted model was subsequently constructed, incorporating lactate, MAP, age (months), duration of mechanical ventilation, and VISmax within the first 24 postoperative hours as covariates to control for potential confounders.
5. Correction for multiple comparisons: For comparisons of the five primary CrSO2 parameters (T1, T2, T3, T4, and CrSO2 min), the Benjamini–Hochberg (BH/FDR) method was applied for correction.

### 3.4 Missing data

This study included a total of 66 pediatric patients who met the inclusion criteria. During the screening process, one patient required ECMO support following surgery and ultimately died of multiple organ failure; this patient was excluded from the final analysis. It was the only excluded patient in this study. Continuous CrSO_2_ monitoring was performed for all patients postoperatively, and data at 6, 12, 24, and 48 h were complete without missing values. All patients remained under continuous monitoring in the Cardiac Intensive Care Unit (CICU) for 2–5 days following extubation, ensuring the validity of 48-hour CrSO_2_ measurements, even among those extubated early. Consequently, no missing data imputation was performed, and statistical analyses were conducted using the complete dataset.

### 3.5 Data review

All children included in this study underwent definitive corrective surgery to establish a biventricular circulation. To ensure pathophysiological consistency within the study cohort, we conducted an independent review of the original surgical records and echocardiographic results for all patients. One case of CAVC and two cases of PA were identified as diagnostic discrepancies in the original records: the children originally diagnosed with CAVC actually underwent complete corrective surgery; one child originally diagnosed with PA actually had severe pulmonary hypertension combined with a VSD and underwent VSD repair, the original diagnostic error originated from the unclear description of the preoperative echocardiogram. Intraoperative exploration confirmed that the pulmonary valve of this child was open but severely damaged; and another child originally diagnosed with PA underwent a right ventricular-pulmonary artery anastomosis, a radical surgical procedure aimed at establishing complete biventricular circulation. All of the above cases fall within the category of biventricular correction, and the relevant diagnoses have been corrected in the table.

After re-including the corrected diagnoses in the analysis, there were no substantial changes in the intergroup comparisons of the primary outcome measures. Before reclassification, the NPOD group accounted for 34 cases (52.3%), and the POD group had 31 cases (47.7%). After reclassification, the incidence rates of the NPOD group and the POD group remained the same as before. Therefore, the core conclusion of this study remains stable after reclassification.

## 4 Results

### 4.1 Comparison of general characteristics

The study included 65 pediatric patients undergoing cardiac surgery with CPB for CHD. Based on the occurrence of POD, patients were divided into NPOD (34 cases, 52.3%) and POD (31 cases, 47.7%) groups. Among paticipants, 45 were male and 20 were female, with a mean age of 3.03 ± 3.11 months, mean height of 59.46 ± 7.73 cm, and mean weight of 5.52 ± 1.98 kg. No statistically significant differences were observed between the groups (*P* > 0.05; Table 1).

**Table 1:** Comparison of general characteristics between the NPOD group and the POD group.

Variable	NPOD group (*n* = 34)	POD group (*n* = 31)	t-value/Z-value	*P*-value
Male/Female (n/%)	23 (67.6)/11 (32.4)	22 (71.0)/9 (29.0)		0.795
Disease Diagnosis				
TOF (%)	14 (41.2)	15 (48.4)		0.392
TGA (%)	4 (11.8)	1 (3.2)		
TAPVC (%)	8 (23.5)	9 (29.0)		
DORV (%)	0 (0)	1 (3.2)		
CAVC (%)	0 (0)	1 (3.2)		
COA (%)	2 (5.9)	2 (6.5)		
VSD (%)	5 (14.7)	2 (6.5)		
PA (%)	1 (3.2)	0 (0.0)		
Cyanotic/Non-cyanotic	26 (76.5)/8 (23.5)	26 (83.9)/5 (16.1)		0.543
Age (months)	30.34	35.92	−1.205	0.228
Height (cm)	59.41 ± 8.49	59.52 ± 6.94	−0.054	0.957
Weight (kg)	5.46 ± 2.19	5.58 ± 1.75	−0.248	0.805

The distribution of diagnoses in the two groups of pediatric patients was as follows: In the NPOD group, the most common diagnosis was TOF (14 cases, 41.2%), followed by TAPVC (8 cases, 23.5%), VSD (5 cases, 14.7%), and TGA (4 cases, 11.8%); In the POD group, the most common diagnosis was TOF (15 cases, 48.4%), followed by TAPVC (9 cases, 29.0%) and COA (2 cases, 6.5%). Based on the characteristics of the surgical outcome, the patients were classified into two groups: cyanotic and non-cyanotic. In the NPOD group, there were 26 cyanotic patients (76.5%) and 8 non-cyanotic patients (23.5%); in the POD group, there were 26 cyanotic patients (83.9%) and 5 non-cyanotic patients (16.1%); the difference between the two groups was not statistically significant (*P* = 0.543). Furthermore, the primary diagnoses in this study were cyanotic CHD representing the majority of cases (80.0%). This indicates that most study subjects were already at high risk for hypoxia, underscoring the clinical relevance of analyzing the relationship between CrSO_2_ and POD.

### 4.2 Association between CrSO_2_ levels and other indicators at different time points and POD

Univariate analysis (Table 2) revealed significant differences in CrSO_2_ levels between the NPOD and POD groups at 6, 12, 24, and 48 h postoperatively (*P* < 0.05), indicating a significant association between early postoperative CrSO_2_ levels and POD. Furthermore, the minimum postoperative CrSO_2_ (CrSO_2_min) in the POD group was significantly lower than that in the NPOD group (*P* < 0.05). In contrast, other indicators reflecting systemic oxygenation, perfusion, and surgical complexity, including SaO_2_, lactate, MAP, VISmax, CPB time, aortic cross-clamp time, operative time, and ICU length of stay, showed no significant differences in univariate comparisons. These indicators, therefore, are more appropriately regarded as background variables reflecting patient severity or as covariates for adjustment in multivariate models.

**Table 2:** Comparison of CrSO_2_ levels and other indicators between the NPOD group and the POD group.

Variable	NPOD group	POD group	t-value/Z-value	*P*-value
T1 CrSO_2_	61.24 ± 13.36	53.52 ± 10.77	2.549	0.013
T2 CrSO_2_	60.47 ± 10.95	53.87 ± 10.82	2.440	0.018
T3 CrSO_2_	38.68	26.77	−2.537	0.011
T4 CrSO_2_	41.21	24.00	−3.669	<0.001
CrSO_2_min	56.62 ± 11.05	49.87 ± 10.19	2.550	0.013
T1 SaO_2_	34.87	30.95	−0.835	0.403
T2 SaO_2_	35.31	30.47	−1.034	0.301
T3 SaO_2_	35.37	30.40	−1.058	0.290
T4 SaO_2_	33.53	32.42	−0.237	0.813
T1 Lactate	33.37	32.60	−0.164	0.869
T2 Lactate	32.03	34.06	−0.435	0.664
T3 Lactate	30.75	35.47	−1.008	0.313
T4 Lactate	31.56	34.58	−0.647	0.517
T1 MAP	64.56 ± 10.92	64.81 ± 8.25	−0.102	0.919
T2 MAP	66.85 ± 10.08	68.00 ± 10.87	−0.442	0.660
VISmax-24h1	30.99	35.21	−0.902	0.367
VISmax-24h2	29.99	36.31	−1.348	0.178
Duration of CPB (min)	102.09 ± 40.93	86.35 ± 40.53	1.555	0.125
Duration of aortic cross-clamping (min)	58.88 ± 32.13	52.48 ± 30.01	0.827	0.411
Operative time (min)	189.41 ± 56.06	173.23 ± 54.53	1.178	0.243
Duration of mechanical ventilation (h)	32.26	33.81	−0.328	0.743
Length of stay in the ICU (h)	31.38	34.77	−0.723	0.469

To control for the risk of false positives associated with multiple comparisons, Benjamini–Hochberg (FDR) correction was applied to CrSO_2_-related comparisons. Results indicated that all CrSO_2_ comparisons remained statistically significant (Table 3), further confirming the robust association between postoperative CrSO_2_ levels and POD.

**Table 3:** Benjamini–Hochberg-corrected results for the CrSO_2_ comparison.

Comparison item	Original *P*-value	BH-corrected *P*-value	Corrected conclusion
T1 CrSO_2_	0.0130	0.0180	Significant
T2 CrSO_2_	0.0180	0.0180	Significant
T3 CrSO_2_	0.0110	0.0180	Significant
T4 CrSO_2_	0.0005	0.0025	Significant
CrSO_2_min	0.0130	0.0180	Significant

### 4.3 Results of multivariate logistic regression analysis

To investigate whether early postoperative CrSO_2_ is an independent predictor of POD, multivariate logistic regression analysis was performed, adjusting for potential confounding variables. Given the limited number of POD events in this study (31 cases), this analysis is explicitly defined as exploratory. The main model included four independent variables, with an event-to-variable ratio (EPV) of 7.75.

Results (Table 4) showed that after adjusting for VISmax within the first 24 postoperative hours, lactate levels at T1, and age in months, a lower CrSO_2_ level at 6 h postoperatively remained an independent risk factor for POD (OR = 0.941, 95% CI: 0.894–0.991, *P* = 0.021). The overall model was statistically significant (*P* = 0.037) and demonstrated good fit according to the Hosmer–Lemeshow test (*P* = 0.132). The remaining variables in the model did not reach statistical significance; however, CrSO_2_ at 6 h postoperatively remained significantly predictive, highlighting its independent predictive value.

**Table 4:** Main model of multivariate logistic regression.

Variable	B	OR	95%CI	*P*-value
T1 CrSO_2_	−0.061	0.941	0.894–0.991	0.021
VISmax in the first 24 h post-surgery	−0.007	0.993	0.942–1.045	0.777
T1 Lactate	0.403	1.496	0.864–2.589	0.150
Age in Months	0.170	1.185	0.943–1.490	0.146
Overall Model (Omnibus)	—	—	—	0.037
Hosmer–Lemeshow	—	—	—	0.132

To assess the robustness of the main model's findings, a series of sensitivity analyses were conducted (Table 5). Specifically, these included: (1) replacing the primary predictor CrSO_2_ at T1 in the main model with CrSO_2_ at T2; (2) replacing it with postoperative CrSO_2_min; and (3) including duration of mechanical ventilation as an additional covariate in the main model. In all sensitivity analyses, the direction of the association between CrSO_2_ and POD remained consistent (all ORs < 1) and statistically significant (*P* < 0.05). These results indicate that the association between low postoperative CrSO_2_ and increased POD risk does not depend on measurements at a specific time point or a particular set of covariates, demonstrating that the findings are robust and reliable.

**Table 5:** Results of logistic regression sensitivity analysis.

Model	Core cerebral oxygenation parameter	OR (95%CI)	*P*-value	Overall model *P*-value
Sensitivity Model 1	T2 CrSO_2_	0.948 (0.900–0.999)	0.045	0.072
Sensitivity Model 2	CrSO_2_min	0.939 (0.889–0.993)	0.026	0.055
Sensitivity Model 3	T1 CrSO_2_ + Ventilation Duration	0.938 (0.891–0.988)	0.016	0.091

### 4.4 Results of LMM analysis

To further investigate the dynamic trajectory of CrSO_2_ in the early postoperative period and its association with POD, a LMM was applied.

#### 4.4.1 Assessment of random effects and model fit

Random effects describe inter-individual variability in the study data. The variance of the random intercept was significant (*σ*^2^ = 16.37, Wald Z = 4.774, *P* < 0.001), indicating substantial heterogeneity in baseline CrSO2 levels across patients. The standard deviation of the random effects was 16.37%, reflecting the magnitude of this heterogeneity. The intraclass correlation coefficient (ICC) was 0.65, indicating that approximately 65% of the total variance was attributable to between-patient differences (Table 6). These findings support the necessity and appropriateness of using LMM for the present analysis.

**Table 6:** LMM results—random effects.

Parameter		Estimate	SE	Wald Z	Significance	95% CI	
						Lower	Upper
Residual		41.91	4.54	9.214	<0.001	33.88	51.85
Intercept [Subject = ID]	Variance	78.15	16.37	4.774	<0.001	51.84	117.83
ICC		0.65					

#### 4.4.2 Fixed-effect analysis: time, POD grouping, and their interaction

The main effect of time was highly significant in both the unadjusted and adjusted models (unadjusted: *F* = 22.099, *P* < 0.001; adjusted: *F* = 20.886, *P* < 0.001; Table 7), indicating that CrSO_2_ levels changed significantly over the 48-hour postoperative period rather than remaining stable.

**Table 7:** Summary of results from the longitudinal LMM for cerebral oxygenation.

Model	Time	POD	Time × POD
Unadjusted LMM	*F* = 22.099, *P* < 0.001	*F* = 9.727, *P* = 0.003	*F* = 0.322, *P* = 0.809
Adjusted AR1 LMM	*F* = 20.886, *P* < 0.001	*F* = 7.595, *P* = 0.008	*F* = 0.433, *P* = 0.730

The main effect of POD grouping was statistically significant in both models (unadjusted: *F* = 9.727, *P* = 0.003; adjusted: *F* = 7.595, *P* = 0.008), suggesting that overall CrSO_2_ levels were consistently lower in the POD group than in the NPOD group, even after accounting for time and random individual variation.

However, the interaction between time and POD grouping was not significant in either model (both *P* > 0.05). This indicates that, although the POD group exhibited lower overall cerebral oxygenation, the temporal pattern of CrSO_2_ change did not differ significantly between groups; POD was therefore primarily associated with a global downward shift in CrSO_2_ levels rather than a difference in trajectory shape.

#### 4.4.3 Between-group comparisons at specific time points based on adjusted marginal means

To further compare between-group differences at various postoperative time points, we calculated the adjusted marginal means at each time point based on LMM models adjusted for covariates and performed simple effects comparisons.

The results show (Table 8) that, after controlling for potential confounding factors, the adjusted mean CrSO_2_ values in the NPOD group remained significantly higher than those in the POD group at T1, T2, and T4 (Bonferroni-corrected *P*-values of 0.009, 0.030, and 0.018, respectively). However, at T3, the difference between the two groups did not reach statistical significance (between-group difference = 4.190, *P* = 0.105). These results suggest that differences in CrSO_2_ in the early (6–12 h) and late (48 h) postoperative periods may be relatively independent of the physiological and treatment-related covariates included in this model, whereas the disappearance of intergroup differences at 24 h postoperatively may be more strongly influenced by factors such as lactate and VISmax.

**Table 8:** Marginal means and simple effects comparison for the adjusted LMM.

Time	Adjusted mean for NPOD	Adjusted mean for POD	Between-group difference	Bonferroni *P*-value
T1	60.070	53.279	6.791	0.009
T2	60.010	54.408	5.601	0.030
T3	64.480	60.290	4.190	0.105
T4	71.153	65.014	6.139	0.018

## 5 Discussion

Using a prospective longitudinal design and combining multivariate logistic regression with LMM, this study demonstrated that low CrSO_2_ levels in the early postoperative period constitute an independent risk factor for POD in children with CHD. This association remained robust even after adjusting for indicators of disease severity, such as VISmax score and lactate levels. Furthermore, the study found that children with POD exhibited persistently lower CrSO_2_ levels, particularly at specific postoperative time points (6 h, 12 h, and 48 h); however, the recovery trend of CrSO_2_ levels in the POD group paralleled that in the NPOD group.

### 5.1 Significance and dynamic changes of CrSO_2_ monitoring in postoperative children

With advances in surgical techniques for CHD, patients undergoing surgery are becoming increasingly younger, and approximately 25% require CPB-assisted surgery during infancy (14). This period coincides with a critical stage of brain development when cerebral vasomotor regulation remains immature (15). Children have limited tolerance to hemodynamic fluctuations, radering than more susceptible to neurological injury (16). In complex CHD such as TOF or TGA, right-to-left shunting and prolonged hypoxemia may further impair cerebral oxygenation and increase the risk of postoperative cerebral hypoperfusion. In this study, NIRS was employed to monitor CrSO_2,_ providing noninvasive, continuous, real-time monitoring of cerebral oxygenation and enabling assessment of the balance between cerebral oxygen supply and demand in critically ill pediatric patients (17, 18). This approach facilitates early detection of cerebral hypoxia and helps prevent irreversible brain injury.

CrSO_2_ undergoes dynamic changes in the early postoperative period (19). This study demonstrated that overall CrSO_2_ levels were significantly lower in the POD group compared to the NPOD group at all observed time points; however, the trends of CrSO_2_ change over time did not differ significantly between groups. This indicates that children with POD experienced an overall downward shift in CrSO_2_ levels rather than delayed recovery. Specifically, between-group differences remained significant at 6 h, 12 h, and 48 h postoperatively, even after adjusting for covariates; however, at 24 h postoperatively, the difference was not statistically significant. The early differences observed at 6 and 12 h may directly reflect the impact of unstable cerebral vascular regulation during anesthesia recovery (19); the diminished difference at 24 h might be attributable to systemic physiological disturbances reaching peak influence on cerebral oxygenation, thus masking POD-related cerebral oxygenation changes (20). The re-emergence of differences at 48 h suggests that if early CrSO_2_ deficits are not effectively mitigated, the effects of inadequate cerebral perfusion may persist (21), closely correlating with the occurrence of POD. This fluctuating pattern (significant in the early phase, unclear in the intermediate phase, and reappearing in the late phase) provides important clinical insights for determining optimal monitoring frequency and key observation periods for CrSO_2_ in pediatric patients undergoing congenital heart surgery.

### 5.2 Predictive role of early changes in CrSO_2_ in POD

In this study, the incidence of POD among pediatric patients undergoing congenital heart surgery with CPB was 47.7%. This rate aligns with those reported by Patel and Meyburg et al. (22, 23), but is higher than that reported by Jian Yang et al. (24) in China. This discrepancy may arise from differences in assessment timing, assessment tools, and patient populations. Despite variations in reported incidence (6), existing evidence collectively indicates that POD is a common and serious postoperative complication in pediatric CHD patients (25, 26), imposing a significant clinical burden. Therefore, it demands heightened attention from healthcare providers, and there is an urgent need to establish standardized assessment protocols for early identification and timely intervention.

In addition to epidemiological characteristics, this study focused on early predictive indicators of POD. A key finding of this study was the identification of early postoperative reductions in CrSO_2_ as an independent risk factor for POD in pediatric CHD patients. Multivariate logistic regression analysis revealed that, after adjusting for VISmax scores, lactate levels at T1, and age in months within the first 24 postoperative hours, lower CrSO_2_ at 6 h postoperatively remained significantly negatively associated with POD. This result suggests that low CrSO_2_ levels are not merely concomitant indicators of critical illness but may independently contribute to the pathophysiology underlying POD. Sensitivity analyses further confirmed the robustness of this finding, suggesting that early CrSO_2_ monitoring could effectively identify pediatric CHD patients at high risk for POD within a critical early postoperative time window.

The brain is a high oxygen-consuming organ, accounting for approximately 20% of systemic oxygen demand (27). In pediatric patients undergoing CPB-assisted cardiac repair, persistently low postoperative CrSO_2_ indicates an imbalance in cerebral oxygen supply and demand, constituting a critical pathological basis for neurological dysfunction and delirium (28). In the study population, 80% were children with cyanotic CHD, who already exhibited chronic hypoxemia and compensatory cerebral vascular changes prior to surgery. Under the dual stresses of CPB and surgery, these patients’ inherently vulnerable cerebral oxygen balance is more readily disrupted, resulting in a greater decrease in CrSO2 and higher susceptibility to POD. This highlights the need to extend clinical attention beyond preoperative and intraoperative periods to include early postoperative phases, which may offer greater dynamic insight and predictive value.

In clinical practice, real-time CrSO_2_ monitoring via NIRS technology enables noninvasive, dynamic evaluation of cerebral oxygenation adequacy, offering objective evidence for early POD detection. Compared to delays inherent in scale-based screening, CrSO_2_ monitoring can identify abnormal cerebral oxygen metabolism before delirium symptoms manifest, thus creating a valuable time window for preventive intervention. This finding supports incorporating CrSO_2_ monitoring into routine postoperative assessment protocols, potentially shifting POD prevention strategies from symptom-oriented approach to one based on physiologically indicators.

### 5.3 Study limitations and future directions

This study has several limitations. First, as a single-center study, the sample size was limited, and no formal *a priori* power calculation was conducted during the design phase; however, *post hoc* power analysis of T1 CrSO_2_ indicated a statistical power of approximately 0.7, below the conventional threshold of 0.8. Therefore, the findings should be considered exploratory evidence supporting the hypothesis. Future studies should perform prospective sample size calculations based on the observed effect sizes and conduct multicenter, large-scale investigations to generate confirmatory evidence. Second, pediatric POD is characterized by symptom fluctuation (7); thus, single assessments may introduce bias. Future research should employ more frequent longitudinal assessments. Third, the current study did not adjust for confounding factors such as parental presence, SvO_2_, and cerebral perfusion pressure; subsequent studies should incorporate these multidimensional variables. Fourth, this study lacked long-term neurodevelopmental follow-up data. Further studies are required to validate the predictive value of early cerebral oxygenation changes for mid- and long-term neurological outcomes. Lastly, to avoid confounding the CrSO_2_ monitoring with postoperative adverse outcomes, this study excluded one child who died after ECMO was initiated postoperatively; this may have affected the estimated incidence of POD. Future studies should include such high-risk children in larger cohorts to validate the generalizability of these findings.

## Data Availability

The original contributions presented in the study are included in the article/Supplementary Material, further inquiries can be directed to the corresponding author.
